# Health Information System Strengthening During Antenatal Care in Haiti: Continuous Quality Improvement Study

**DOI:** 10.2196/55000

**Published:** 2024-06-14

**Authors:** Meredith Casella Jean-Baptiste, Thamar Monide Vital Julmiste, Ellen Ball

**Affiliations:** 1 Hôpital Universitaire de Mirebalais Sante Fanm Mirebalais Haiti; 2 Partners In Health Boston, MA United States

**Keywords:** maternal health, health informatics, quality improvement, Plan-Do-Study-Act, PDSA, maternal, neonatal, data collection, prenatal, outpatient, electronic data, nursing, nursing staff, nursing leadership

## Abstract

**Background:**

Journey to 9 Plus (J9) is an integrated reproductive, maternal, neonatal, and child health approach to care that has at its core the goal of decreasing the rate of maternal and neonatal morbidity and mortality in rural Haiti. For the maximum effectiveness of this program, it is necessary that the data system be of the highest quality. OpenMRS, an electronic medical record (EMR) system, has been in place since 2013 throughout a tertiary referral hospital, the Hôpital Universitaire de Mirebalais, in Haiti and has been expanded for J9 data collection and reporting. The J9 program monthly reports showed that staff had limited time and capacity to perform double charting, which contributed to incomplete and inconsistent reports. Initial evaluation of the quality of EMR data entry showed that only 18% (58/325) of the J9 antenatal visits were being documented electronically at the start of this quality improvement project.

**Objective:**

This study aimed to improve the electronic documentation of outpatient antenatal care from 18% (58/325) to 85% in the EMR by J9 staff from November 2020 to September 2021. The experiences that this quality improvement project team encountered could help others improve electronic data collection as well as the transition from paper to electronic documentation within a burgeoning health care system.

**Methods:**

A continuous quality improvement strategy was undertaken as the best approach to improve the EMR data collection at Hôpital Universitaire de Mirebalais. The team used several continuous quality improvement tools to conduct this project: (1) a root cause analysis using Ishikawa and Pareto diagrams, (2) baseline evaluation measurements, and (3) Plan-Do-Study-Act improvement cycles to document incremental changes and the results of each change.

**Results:**

At the beginning of the quality improvement project in November 2020, the baseline data entry for antenatal visits was 18% (58/325). Ten months of improvement strategies resulted in an average of 89% (272/304) of antenatal visits documented in the EMR at point of care every month.

**Conclusions:**

The experiences that this quality improvement project team encountered can contribute to the transition from paper to electronic documentation within burgeoning health care systems. Essential to success was having a strong and dedicated nursing leadership to transition from paper to electronic data and motivated nursing staff to perform data collection to improve the quality of data and thus, the reports on patient outcomes. Engaging the nursing team closely in the design and implementation of EMR and quality improvement processes ensures long-term success while centering nurses as key change agents in patient care systems.

## Introduction

### Background

Journey to 9 Plus (J9) is a comprehensive reproductive, maternal, neonatal, and child health (RMNCH) approach to care that has at its core the goal of decreasing the rate of maternal and neonatal morbidity and mortality in Haiti. According to the World Bank, maternal mortality in Haiti is 350 per 100,000 live births [1] and neonatal mortality is 24 per 1000 live births [2]. To show this program’s impact, data collection must be of the highest quality to be of value. The J9 program was initiated in July 2018 at Hôpital Universitaire de Mirebalais (HUM), a Zanmi Lasante (ZL) and Partners In Health (PIH)–supported tertiary referral hospital located in the Central Plateau of Haiti. The 4 pillars of this RMNCH approach encompass group antenatal care; group pediatric appointments; home visits; and psychosocial screening, counseling, and support. The J9 program serves women and their families in the Mirebalais catchment area, which has a population of 97,755 persons [3]. The team that initiated J9 at HUM, primarily staffed and run by nurses, helped to improve and expand the data capture within the existing electronic medical records (EMRs) on the OpenMRS [4] data collection platform. This continuous quality improvement (CQI) project was conducted with the PIH health information system (HIS) team located in Boston, Massachusetts.

### Problem Description

Working in rural Haiti, where there is limited access to EMRs, means monthly reports depend on paper-based patient charts and registers transferred into electronic forms. Depending on the quality of the transfer of these data, values are sometimes not transcribed correctly from the paper register format. At other times, physical charts go missing along with entire patient histories, including years of laboratory results, prescription history, documentation of illnesses, hospitalizations, surgical interventions, and outpatient follow-up visits, leaving health care professionals, including nurses, midwives, and doctors, with only the subjective history of the patient to rely on for continued care. This is not only frustrating for patients and medical providers but can impact wait times, contribute to medication errors, and can compromise patient health and safety; it does little in terms of assuring quality of care [5]. The loss of prenatal, postpartum, or pediatric patient charts in addition to the use of paper registers contributed to incomplete and inconsistent reports. The medical provider had to start a new empty paper chart each time a patient’s paper chart was lost or not located at the time of the visit. The ideal solution would be to enter all outpatient prenatal visit data into an EMR. At HUM, the OpenMRS EMR was not consistently being used by medical providers, including the J9 staff. A quality improvement project was initiated to address the gaps in the system and to ensure more consistently complete and high-quality reports.

### Available Knowledge

A review of the literature on the role and impact of EMRs in data collection in low- and middle-income countries (LMICs) found that a robust EMR system is critical in RMNCH services to ensure quality of the data collected, thereby enhancing the quality of care provided to this vulnerable group [6]. Additionally, social needs can be assessed and managed, particularly for vulnerable populations, by using EMRs in clinical settings [7]. HISs have the potential to improve the quality of care provided to patients by increasing adherence to guidelines, enhancing disease surveillance, and decreasing medication errors [7-10].

An EMR system provides important health and safety benefits [8]. In LMIC settings, it has been shown to reduce wait times and medication order errors [11]. However, experience from global health informatics projects shows the need for a thorough understanding of the local environment and populations in low-resource settings, such as those in LMICs [10,12]. Despite challenges facing LMICs, studies have shown how EMRs can succeed with support if an EMR is designed and implemented to fit into local environments [13]. Additionally, routine data quality assessments of EMRs have been associated with improvements in data quality in LMICs [14], with timeliness, availability of data for health reports, and staff satisfaction with an electronic system compared to paper-based systems [15]. Using health management information system data is an important part of the CQI process and leads to improving data quality [16].

### Rationale

To our knowledge, this is the first study to describe the use of CQI methodology to enhance EMR data quality and use and improve the quality of care in maternal and newborn services in a comprehensive maternal and newborn care program in rural Haiti. Data collected through an EMR are more accessible and reliably stored. A closer examination of the J9 program exposes how point of care data entry into an EMR-based system can be advantageous for numerous reasons in the context of rural Haiti. For example, critical patient data, such as prenatal risk factors, history, blood pressure, other vital signs, laboratory results, and syphilis/HIV status, are all essential in the provision of continuity of care. Prenatal care optimizes both the outcomes and experiences of maternal health care for pregnant women along with outcomes for newborns [17].

Access to paper-based patient charts at each prenatal visit for documentation as well as during any hospitalization or at the time of delivery and postpartum is essential for point of care decision-making in cases of prevention of vertical disease transmission, preeclampsia diagnosis, and treatment of infections, and ensures compliance with updated recommendations and high quality of care. The loss of data through a paper-based system when charts are missing or lost during visits or at the time of delivery highlights the gaps in the continuity of care that can increase the risk of lack of treatment or follow-up for previously detected illnesses.

Monthly reports from paper charts and registers were transcribed into an electronic format to track key indicators. This exposed the challenges and potential data loss of J9 maternal and newborn health outcomes. Improved electronic data collection at point of care, including laboratory results, translates to easier storage for analysis, reporting, and follow-up care, thereby impacting quality of care. This translates to reports that can illustrate the effects of an accompaniment model and its impact on maternal and neonatal morbidity and mortality. An EMR system can contribute to the improvement of data quality; however, reliable internet access, motivated staff willing to enter the data in the EMR system, availability of hardware and appropriate software, and updates to the system to adapt to the needs of the local team are all essential to an operational system [11,13,15].

At the start of the improvement project at HUM, a major challenge was that the EMR had the capacity to collect only half of the essential prenatal data during a point of care visit compared to the data collected on paper charts. This was due to the launch of the EMR before it met all the needs of the team on the ground, resulting in the staff having to double chart (both on paper and electronically). Double charting is time-consuming, can contribute to inconsistencies [15], and can create staff and patient frustrations, as the workflow is significantly impacted. However, this is reflective of the journey from paper to electronic systems in many underresourced settings, where paper-based patient charts are the norm and monthly reports are compiled in an electronic format, causing duplication and overlap of data reporting [12,15,18].

### Specific Aims

This study aimed to improve the electronic documentation of outpatient prenatal care in the HUM EMR by J9 staff from 18% to 85% between January and September 2021. The team fixed 85% as the target for the objective of this CQI project for 2 reasons. First, we wanted an attainable goal that, upon success, would motivate the team to continue to improve, thus creating a sustainable result. Second, the EMR system is down on occasions, thereby preventing a goal of 100%. The data from the backup system of paper charts in these cases are not always retrospectively entered into the EMR following visits. The experiences that this quality improvement project team encountered could contribute to the literature in terms of ways to improve electronic data collection as well as the transition from paper to electronic documentation in a burgeoning health care system.

## Methods

### Study Approach

CQI is described as a systematic and structured approach to improving an identified process or service, consisting of continuous feedback loops with outcomes measured and evaluated throughout the process [19]. CQI is both a culture and a methodology that improves work processes, ensuring better outcomes. It is a philosophy that encourages all project team members to continuously ask how they can improve their work systems and is often associated with methodologies like Plan-Do-Study-Act (PDSA) cycles [20]. Compared to research, CQI does not aim to create new generalizable knowledge; however, both quality improvement and research require a consistent methodology. CQI intersects with research in the process to iteratively test conditions required to adapt published research findings to local context [21].

### Context

A CQI strategy was undertaken to improve the collection of electronic data being entered into the EMR system at HUM. The team used several CQI tools to conduct this project, including baseline data collection, a root cause analysis using the Ishikawa diagram, the Pareto diagrams, PDSA improvement cycles to document incremental changes, and analysis of the results of each change. This quality improvement project team was led by TMVJ, a nurse leader at HUM, and a J9 program coordinator. Other team members included a midwife (MCJ-B), nurse (Jovana Altenor), HIS team members in both Boston (EB) and Haiti (Jimmy Jean-Baptiste), as well as a pediatrician (Michel-Ange Hilaire). All CQI project team members are also involved in J9 activities and are invested in the success and sustainability of the program.

### Ethics Approval and Consent to Participate

All methods of quality improvement were performed in accordance with the regulations for human subjects research protections according to the Department of Health and Human Services (45 Code of Federal Regulations (Common Rule) part 46) [22]. As this was a quality improvement project, it did not involve any direct patient contact, and all patient information in the data sets generated for the analysis was retrospective, deidentified, and anonymized prior to analysis. For these reasons, informed consent was waived for the purposes of this study. The original informed consent allows the secondary analysis without additional consent. The ethics committee that reviewed and approved the study protocol and waived the need for informed consent was the ZL institutional review board in Haiti (IRB application number ZLIRB11242022). In addition, approval for this CQI project was obtained by the Quality Improvement Committee of the Hôpital Universitaire de Mirebalais in Haiti. As the data analyzed were retrospective, no compensation was offered or received in this study.

### Intervention

**Figure 1:**
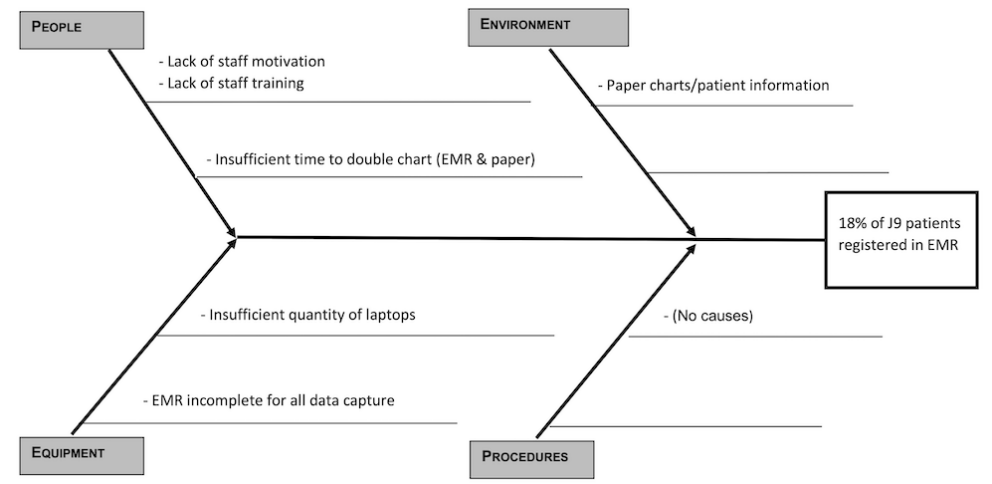
Ishikawa diagram of cause and effect of maternal health antenatal electronic medical record data entry in the quality improvement project at Hôpital Universitaire de Mirebalais (Haiti) in 2020-2022. EMR: electronic medical record; J9: Journey to 9 Plus.

The quality improvement team used the standard quality improvement tools, as outlined by the Institute for Healthcare Improvement [23], to improve the data entry into the EMR. The challenges faced in J9 were identified when a root cause analysis was performed at the start of this CQI project. These key causes contributed to the baseline evaluation; only 18% of the prenatal documentation of patient visits were being entered into the EMR [24]. The Ishikawa or “fishbone” diagram (Figure 1) visibly displays and helps teams understand the multiple root causes that contribute to an effect and highlights areas for improvement [23]. The team members identified and classified the causes under 4 categories: people, environment, equipment, and procedures.

**Figure 2:**
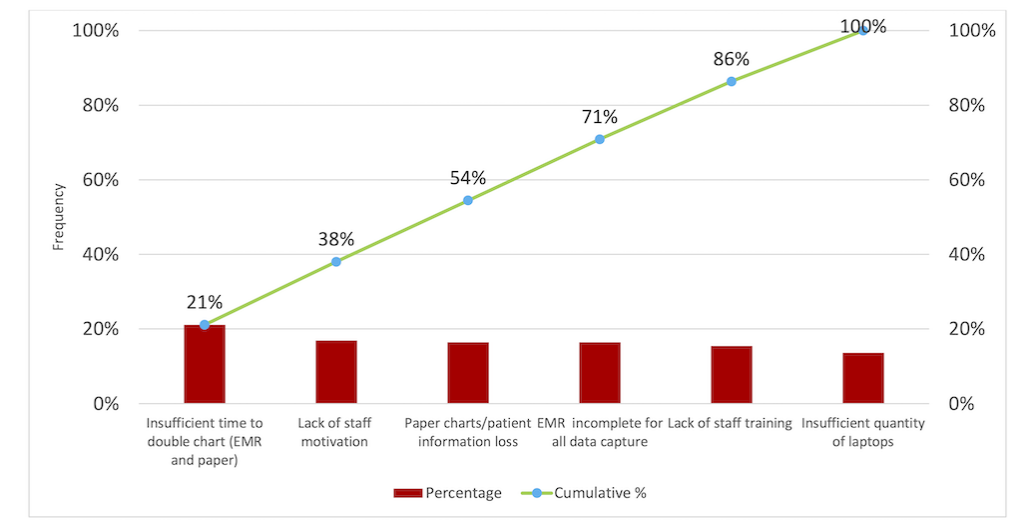
Pareto diagram of maternal health antenatal electronic medical record data entry in the quality improvement project at Hôpital Universitaire de Mirebalais (Haiti) in 2020-2022. EMR: electronic medical record.

The Pareto diagram (Figure 2) was created using the causes identified in the Ishikawa diagram. According to the Institute for Healthcare Improvement, a Pareto diagram is a chart in which the various factors that contribute to an overall effect are arranged in the descending order of importance according to the magnitude of their effect. This ordering helps a CQI team identify the vital few factors and helps a team concentrate its efforts on the factors that have the greatest impact. It also helps a team communicate the rationale for focusing on certain areas [23]. For the purposes of this CQI project, the team calculated the mean percentage for each cause or factor that contributed to the low amount of prenatal data being entered into the EMR. The score was based on interviews with all members of the J9 team, who were asked to rank the contributing factors that prevented them from entering the point of care data into the EMR on a scale of 1-10, with 10 being the factor that most contributed to the lack of data entry. Insufficient time to double chart received an average of 9/10; lack of staff motivation, paper chart/information loss, the EMR platform being incomplete, and lack of training were all ranked at 7/10; and insufficient laptops was ranked at 6/10.

According to the 80/20 rule, whereby resolving 20% of the root causes will resolve 80% of the effects [23], the team was able to then focus on the vital few factors that made up 20% of the causes. In this case, the team addressed the root cause as “insufficient time to double chart” first. It is worth noting that the Pareto diagram does not account for hidden factors such as power outages, wireless/internal EMR access issues, deficiencies in the user interface, user familiarity over time with electronic data entry, stress of sociopolitical unrest on staff, or exceptionally busy days that throw off normal patterns.

### Measures

The indicator identified for this quality improvement project was the percentage of pregnant women enrolled in the J9 program whose data were completely entered in the EMR system from November 2020 through September 2021. The numerator was the total number of pregnant women enrolled in J9 whose prenatal visit data were completely entered in the EMR, and the denominator was the total number of unique pregnant women enrolled in J9 who checked in for their visit, captured through the EMR. Results were tracked for sustainability through April 2022. The team fixed 85% as the objective for this project. The team reviewed the following elements, which were analyzed for improvement: (1) vital signs; (2) gravidity, parity, abortion, living (GPAL); (3) last menstrual period (LMP); (4) fetal heart tone (FHT); and (5) fundal height (FH). If entered, these indicate that the prenatal record was considered complete.

These data were captured monthly for the project’s duration with the help of the monitoring and evaluation team at Hôpital Universitaire de Mirebalais. The data indicator of LMP was chosen to indicate completeness because only the J9 staff would have entered this information in the EMR and at that time; LMP did not carry over to the next prenatal visit. The J9 staff interventions (completeness of prenatal data entry) would be affected by the interventions taken to improve this indicator and thereby directly impact the success of this CQI project.

## Results

### Baseline Evaluation

**Figure 3:**
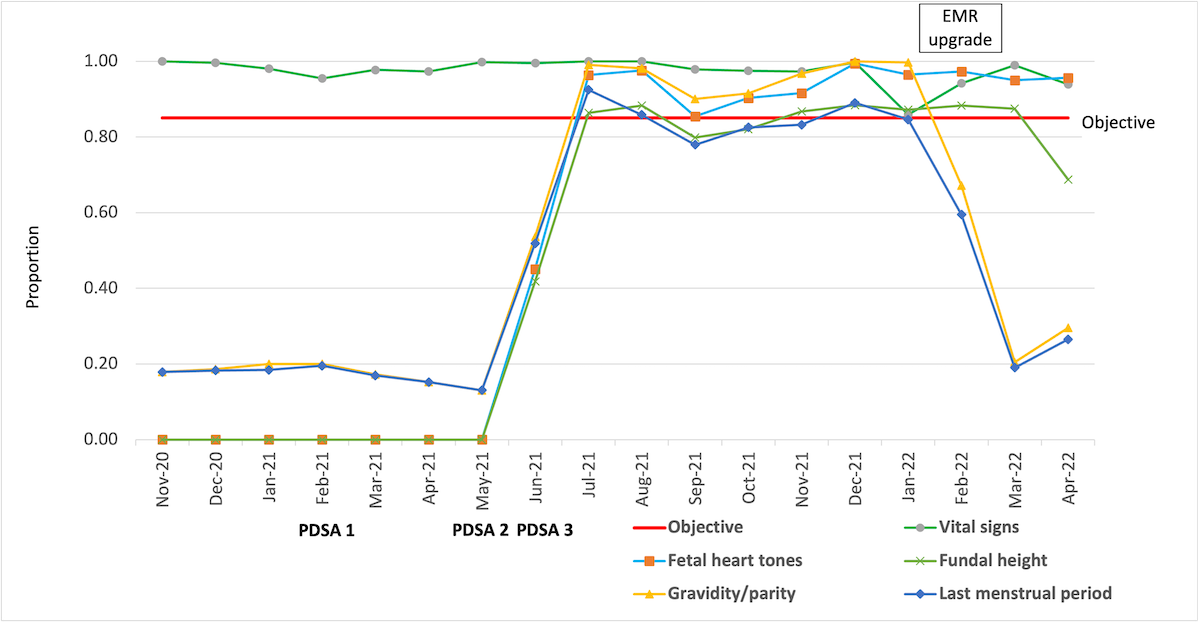
Run chart diagram showing all Journey to 9 Plus maternal health antenatal visit results in 2020-2022 in the Hôpital Universitaire de Mirebalais (Haiti) electronic medical chart quality improvement project. In February 2022, the electronic medical record system upgrade allowed last menstrual period and gravidity/parity data to be only entered on the initial antenatal intake. These data were maintained throughout the pregnancy. EMR: electronic medical record; PDSA: Plan-Do-Study-Act.

The initial evaluation found 18% (58/325) of the pregnant women enrolled in J9 whose GPAL and LMP were entered at their prenatal visit in the EMR in November 2020 [24]. At this point in time, the EMR could not capture FHT or FH; therefore, these data points could not be considered in the completeness of the data entry. Other data entry points at the start of the project were as follows: vital signs, 100% (325/325); FHT, 0% (0/325); and FH, 0% (0/325), at the start of the project (see Baseline Evaluation [November 2020] in Figure 3).

When vital signs were analyzed, they were consistently entered for 97% (301/309) of all J9 prenatal patient contacts throughout the entire project. However, GPAL and LMP, if entered, were consistently together. Other variables such as high-risk pregnancy diagnosis, medication prescription, pregnancy history, and family history were entered as needed.

### Analysis of Improvement

A total of 17,404 outpatient women’s health visit records were extracted from the EMR from November 2020 through April 2022. Of these, 10,524 were prenatal patient visit records. Roughly, half of the prenatal visits or 5561 point of care contacts were with patients enrolled in J9 and were included in the analysis. The 4963 patient contacts with patients not enrolled in J9 were excluded from the analysis. An average of 309 (200-422) J9 prenatal check-ins were recorded each month in the EMR for the project duration [24]. The baseline evaluation showed that 18% (58/325) of the LMP and GPAL electronic data were captured during prenatal visits at the start of the CQI project. The EMR did not collect FHT or FH data at the beginning of this project. To ensure sustainability of the changes, the team continued to measure the data being entered at point of care delivery and found the following: (1) vital sign data continued to be entered 95% (301/309) of the time; (2) FHT and FH data improved from 0% (0/325; November 2020 through May 2021) to 95% (286/304), and 84% (272/304) of FHT and FH data, respectively, were entered at prenatal visits starting in July 2021.

In February 2022, the EMR was upgraded for user experience. The improvement allowed LMP and GPAL data to be entered only at the first antenatal visit and not at follow-up visits, as these data points would not change throughout pregnancy. The run chart diagram (see Figure 3) shows an improvement in LMP and GPAL from 18% (58/325) to 91% (310/341) of prenatal contacts, dropping off drastically to 24% (52/215) of prenatal visits starting after the EMR system upgrade. This is a positive reflection of data for LMP and GPAL no longer being required for follow-up antenatal visits.

The team achieved their initial goal of 85% of J9 prenatal visits encounters having electronic data entry into the EMR because of the interventions described in the PDSA cycles, which depended upon strong collaborative teamwork. The amount of data lost when using paper charts can be significant. The results from the first and second PDSA cycles show minimal change from baseline. However, the third PDSA cycle addressed the completeness of the maternal child health EMR, and therefore, the concerns of double charting that were expressed by the staff from the beginning (see Table 1). The use of both paper and electronic charts was necessary when the EMR was incomplete. With the HIS system upgrade to include all of the data points prioritized by the HUM providers on the ground as feedback during the interprofessional Friday meetings, the Boston and Haiti HIS/monitoring and evaluation teams were able to augment the OpenMRS EMR system to suit the needs of the providers on the ground. This project also considered the required reporting indicators that are compiled each month when assembling the monthly report.

**Table 1:** Plan-Do-Study-Act cycles of improvement of maternal health antenatal electronic medical record data entry in the quality improvement project at the Hôpital Universitaire de Mirebalais (Haiti) in 2020-2022.

	Plan	Do	Study	Act
December 2020: first PDSA^a^	Addressed the problem of lack of staff motivation to enter data into the EMR^b^	Encouraged the J9 providers, 4 nurses and 1 nurse midwife, to enter the patient prenatal information at point of care directly into the EMR system instead of onto the paper forms. Inaccessibility of the EMR system due to connectivity issues was a primary concern expressed; decision to use paper forms only when the system was down resolved this issue.	This cycle resulted in an increase from 18% to 20% data entry in February 2021 (see results in Figure 3).	The team decided to adapt the strategy and continue with the second cycle.
May 2021: second PDSA	Addressed the lack of staff training on EMR use; training was held on EMR data entry for the staff	ZL^c^-based HIS^d^ team conducted an EMR-specific training on May 20-21, 2021, for the J9 staff.	This resulted in an increase from 13% (55/422) of prenatal visits entered in May 2021 to 52% (206/397) of prenatal J9 electronic data entered in June 2021.	The team decided to adapt the strategy and continue with the third cycle.
April-June 2021: third PDSA	Addressed the root causes “insufficient time to double chart” and “incomplete EMR platform.” A team of ZL medical leadership, J9 staff, M&E^e^, and HIS staff in both Haiti and Boston worked together to address EMR issues.	Addressed the needs of outpatient women’s health staff at HUM^f^, the hospital reporting indicators, and the OpenMRS system across the ZL network of hospitals and clinics in Haiti who would be using the platform for data collection and interpretation. Weekly team meetings were conducted to satisfy the needs of the stakeholders and to ensure quality data collection.	The results following this cycle showed an average of 89% (272/304) of J9 prenatal patient data entered completely in the EMR.	The changes were adopted. The results have been sustained, and the team continues to work without paper charts, unless the electronic system is inaccessible and backup paper forms are necessary. The results of this third PDSA cycle and the sustained success can be seen in Figure 3.

^a^PDSA: Plan-Do-Study-Act.

^b^EMR: electronic medical record.

^c^ZL: Zanmi Lasante.

^d^HIS: health information system.

^e^M&E: monitoring and evaluation.

^f^HUM: Hôpital Universitaire de Mirebalais.

## Discussion

### Principal Results

An EMR system can contribute to a reduction in maternal and neonatal deaths through the collection of high-quality data that can facilitate follow-up and recommendations and adherence to guidelines [7-10]. According to the J9 approach to maternal, neonatal, and pediatric group care, whose objective is to reduce the rate of maternal and neonatal deaths, an EMR system is essential.

Point of care data entry of prenatal medical information directly into the EMR system was performed by existing staff, consisting of 4 nurses and 1 nurse-midwife. No additional staff were hired to assist with the EMR data entry. When the EMR system was down, the staff used paper charts as backup and later entered the information into the system. This data entry following the appointment is time-consuming and not always completed, thereby explaining why the team did not reach 100% electronic data entry. The team hopes that the use of the EMR and the capability to sustain complete, high-quality data will therefore reduce staff time compared to recording, reporting, and searching through paper charts for specific patient information or care received. Transitioning from retrospective to point of care data entry has been a significant hurdle to overcome.

The biggest improvements were based on the enhanced clinical staff interface for data collection (PDSA 3) because it reduced the amount of time spent on data entry and allowed for one system for data collection. Importantly, the staff were willing to transition from double entry to prioritizing the EMR data collection. This highlights that nurses value a stable point of care system with high-quality data capture, which has ultimately driven our transition. The expectation now is to continue providing significant benefits for J9 patients, staff, and the organization to help sustain these improvements.

An effective EMR implementation could confer cost savings [10], even in a low-resource setting, if it supports good documentation and access to key clinical data [25]. These savings could improve health care efficiency and safety, and HIS-enabled prevention and management of chronic disease could eventually double those savings while increasing health and other social benefits [10]. Cost savings can also be considered through reduction of medication errors and adverse drug events in ambulatory settings and reduction in repeat laboratory tests [25] and duplicate consultations. Studies have shown improved patient safety from EMR use in hospitals and ambulatory care that focuses on alerts and reminders [10].

The lessons learned from this team experience are valuable for others to also learn from and can be applied in a variety of settings. The work that we have conducted for J9 data collection is being used in other health facilities in Haiti in addition to the Maternal Center of Excellence in Sierra Leone. The primary lesson is that a fully integrated EMR point of care system eliminates the need to double chart and improves data quality. The EMR system is mature now after numerous rounds of feedback from the J9 clinical team and software iterations. Since OpenMRS and the antenatal configuration is open source, free, and multilingual, it is available to all worldwide.

The outcomes of this CQI project came about as a result of a great amount of teamwork and collaborative efforts to achieve the outcomes desired. A CQI project requires team members taking the roles of leader, facilitator, secretary, timekeeper, and members. Beyond nursing, this interdisciplinary project involved physicians, monitoring and evaluation teams, EMR trainers, and information technology software developers. This cross-collaborative approach was critical to achieving the project goals.

Nursing implications included having a strong and dedicated nursing leadership to perform the paper to electronic transition in addition to nursing staff motivated to improve data collection. These factors contributed to the improvement of the quality of the J9 data set and, therefore, improvement in the quality of the monthly reports on patient outcomes. EMR-based quality improvement projects can succeed when nurses lead and are involved in the process, resulting in the success and sustainability of the outcome.

Other technical solutions to aid this transition to getting closer to 100% of data entry include a solution that has been adapted in other resource-poor settings where OpenMRS is being used. Optical character recognition software has been used to scan documents [11,26,27], which could potentially assist the staff through back-entering the paper forms when the EMR is unavailable. Additionally, in Nigeria, a model of information system adoption looked at system usability, effort expectancy, and net benefits, showing significant results when system changes are made in terms of quality of the interface, with higher staff satisfaction and a significant influence on use [28].

Although our project was conducted in only 1 health facility, our experiences are reflective of what happens in many low-resource settings in terms of effectiveness of interventions that increase EMR data entry in LMICs using OpenMRS. If this project had occurred in a much better resourced facility than is typical in Haiti, then the results may not have been generalizable. However, our project was set in a low-income setting and is reflective of the environment, staff, and other resources typically available in the country; it is for this reason that we propose that the results are more likely to be generalizable.

### Limitations

Only LMP, estimated date of delivery, and GPAL data were entered during intake at the first prenatal visit since February 2022. Elements such as family history and pregnancy history are only sporadically entered during the first prenatal visit, and these indicators can be improved upon. The first prenatal visit data were not analyzed as part of this paper, as the first prenatal visit was performed prior to registration in the J9 program. Additionally, the EMR system was down on occasion, and although there was a backup system of paper charts, missing data were not back-entered into the EMR.

Lessons learned on the part of the staff and organization from this experience highlight the importance of a core group of dedicated medical providers to communicate and collaborate with HIS staff who have the desire and bandwidth to take on the challenge of helping a university hospital in Haiti to make the transition from paper-based records to EMRs. Without this commitment to the objective of transitioning from paper to electronic data collection, the team would not have been able to span the digital divide [29] and achieve the sustained results such as this team observed. Staff in clinics or hospitals in low-resource settings may not have the bandwidth to even collect point of care electronic data, thereby impacting their ability to share successes, challenges, and needs through reporting mechanisms.

Other sequelae of this scale-up include a positive subsequent influence on point of care data entry during the first and follow-up individual prenatal visits, gynecologic visits, and family planning visits outside of the J9 program. These data were not included in this paper, but there has been significant uptake of the EMR system among medical providers across all outpatient women’s health services. These positive benefits seen in J9 and outpatient women’s health services are helping to sustain the process for getting over the hump of point of care data entry.

It is important to note that the women’s health outpatient department is the first of the many services in the hospital to make the transition from paper forms to electronic forms. The team has also encountered challenges in hospitalization transition, such as the labor and delivery services, in adopting point of care electronic documentation, where timing and access to quality data are even more important.

Lastly, challenges around the need for additional laptops were identified in the Ishikawa diagram, but it has not been completely addressed. The current desktop hardware system is aging; resolving procurement issues to update and sustain the EMR system is another challenge that the team has faced.

### Conclusion

The J9 CQI project demonstrated improvement in the electronic documentation of outpatient antenatal care from 18% (58/325) to 89% (272/304) into the HUM EMR by J9 staff from January to September 2021. This project comprised 3 PDSA cycles; however, it was found that the most significant improvements emerged from the enhanced EMR interface for data collection (PDSA 3). This change reduced the amount of time staff spent on data entry, which facilitated the transition from double entry to prioritizing the EMR for point of care data collection. This project also highlights the value nurses place on a stable point of care system with high quality data capture, which ultimately drove our transition.

This is an example of strong empowered hospital nursing leadership taking the initiative to invest time and energy into its nursing staff to improve antenatal data collection and to make the transition from paper to an EMR system. Implicit in this work is the notion that the clinicians—primarily nurses in this case—are both the lead beneficiaries (along with the patients) of timely access to high quality data as well as the ones doing all or most of the work in data collection and entry. Other beneficiaries such as hospital leadership, management, and funders do not directly contribute to the work but help in supporting systems and hardware. For this reason, involving the nursing team closely in the design and implementation of the EMR and quality improvement processes supports the centrality of nurses as key actors and workers.

It is important to note that as stated above, in most resource-limited settings like Haiti, access to electronic data collection is limited or incomplete and often duplicate and overlapping, thereby increasing the workload for health workers [12,18]. High-quality, timely, and complete data entry in the EMR supports clinical care improvement and reduces the need for duplicate data collection and the effort on reporting tasks. The ultimate success of this project was due to the CQI project leadership, nursing leadership, and the diverse skill sets of the members of the project, all of whom impacted the long-term sustainability of the results, and on the quality of documentation in the outpatient women’s health services at HUM. These results can, in turn, improve patient quality of care. The experiences that this quality improvement project team encountered can contribute to the literature through the strategy undertaken by this team to improve and sustain electronic data collection as well as the transition from paper to electronic documentation within a burgeoning health care system.

## Abbreviations

CQI: continuous quality improvement
EMR: electronic medical record
FH: fundal height
FHT: fetal heart tones
GPAL: gravidity, parity, abortion, living
HIS: health information system
HUM: Hôpital Universitaire de Mirebalais
J9: Journey to 9 Plus
LMIC: low- and middle-income country
LMP: last menstrual period
PDSA: Plan-Do-Study-Act
PIH: Partners In Health
RMNCH: reproductive, maternal, neonatal, and child health
ZL: Zanmi Lasante

## Data Availability

The deidentified data sets that were generated from the Hôpital Universitaire de Mirebalais electronic medical records in Haiti and analyzed during this study are not publicly available. All relevant data are within the manuscript.
